# High Level Ethanol from Sugar Cane Molasses by a New Thermotolerant *Saccharomyces cerevisiae* Strain in Industrial Scale

**DOI:** 10.1155/2013/253286

**Published:** 2013-12-01

**Authors:** M. Fadel, Abeer A. Keera, Foukia E. Mouafi, Tarek Kahil

**Affiliations:** ^1^Microbial Chemistry Department, National Research Center, Dokki, Cairo, Egypt; ^2^Genetic Engineering and Biotechnology Division, Microbial Biotechnology Department, National Research Center, Dokki, Cairo, Egypt

## Abstract

A new local strain of *S. cerevisiae* F-514, for ethanol production during hot summer season, using Egyptian sugar cane molasses was applied in Egyptian distillery factory. The inouluum was propagated through 300 L, 3 m^3^, and 12 m^3^ fermenters charged with diluted sugar cane molasses containing 4%-5% sugars. The yeast was applied in fermentation vessels 65 m^3^ working volume to study the varying concentrations of urea, DAP, orthophosphoric acid (OPA), and its combinations as well as magnesium sulfate and inoculum size. The fermenter was allowed to stay for a period of 20 hours to give time for maximum conversion of sugars into ethanol. *S. cerevisiae* F-514 at molasses sugar level of 18% (w/v), inoculum size of 20% (v/v) cell concentration of 3.0 × 10^8^/mL, and combinations of urea, diammonium phosphate (DAP), orthophosphoric acid (OPA), and magnesium sulfate at amounts of 20, 10, 5, and 10 kg/65 m^3^ working volume fermenters, respectively, supported maximum ethanol production (9.8%, v/v), fermentation efficiency (FE) 88.1%, and remaining sugars (RS) 1.22%. The fermentation resulted 13.4 g dry yeast/L contained 34.6% crude protein and 8.2% ash. By selecting higher ethanol yielding yeast strain and optimizing, the fermentation parameters both yield and economics of the fermentation process can be improved.

## 1. Introduction

Yeast selection for fuel ethanol production over the past two decades, most bioethanol related researches in developing tropical countries have focused primarily on the isolation of local *Saccharomyces* yeasts and their use for industrial ethanol production [1–6]. Yeasts have been isolated from many sources for industrial purposes. Such sources include cashew, apple juice [7–9], and fermenting cassava tubers [10] among others. Despite the evolving trend of using bacteria for ethanol production, yeast is still the primary choice for fermentation [11]. Yeasts are used in the fermentative production of ethanol, alcoholic beverages, baking products, protein, and vitamin supplements in human and animal diets as well as in the production of single cell proteins. However, efforts to characterize these yeasts have fallen short of expectation. In the assessment of yeasts of the genus *Saccharomyces* for economic and efficient ethanologenic processes, certain specific physiological properties are important and required. These include good tolerance to high concentrations of ethanol, sugars, and acids as well as high osmotic pressure [12–16]. Also good flocculation/sedimentation ability depending on process requirements as well as good invertase activity and excellent specific ethanol productivity is important characteristics of yeasts capable of converting sucrose to ethanol [17]. This paper reports the results of a study based on the comparative analysis of ethanol production along with byproducts commercial yeast strains in a local distillery of Egypt.

## 2. Material and Methods

### 2.1. Sugarcane Molasses

Sugarcane molasses procured by Egyptian Sugar and Integrated Industries Company is used as carbon source for ethanol production in the Distillation Factories, El-Hawamdia, Giza, Egypt.

### 2.2. Yeast Strain

*Saccharomyces cerevisiae* F-514, which isolated by the first author was obtained from Microbial Chemistry Lab. National Research Centre, Dokki, Cairo Egypt. 

### 2.3. Inoculums Preparation

Sterilized 500 ml capacity conical flasks each contained 200 ml of medium containing (g/L) malt extract, 3, yeast extract, 3, peptone, 5 and sucrose, 30 was steam sterilized at 121°C for 15 minutes. Cooled to room temperature, then inoculated with a loop of yeast strain * S. cerevisiae* F-514 and incubated statically at 34°C for 24 hrs, then transferred to flat round bottom flasks of 2 L capacity each containing 1L sterilized molasses diluted to 4-5% (w/v) sugar content supplemented with 0.4% DAP and 0.2% yeast extract. The inoculated flat round bottom flasks are incubated statically at 34°C for 24 hrs.

Yeast cultures were prepared in separate seed fermenters of 300 L capacity. Molasses diluted to 4-5% (w/v) sugars content was supplemented with Urea (0.1%, and 0.2% DAP w/v, pH of the medium was adjusted to 4.6 (Pre optimized) using diluted NaOH and diluted H_2_SO_4_. The medium was steam sterilized. After cooling to 32°C ±2 two flat round bottom flasks from above inoculum strain of yeast were added and the seed fermenters were aerated to facilitate the growth of yeasts. At the end of first stage of 16 hours of continuous circulation, sample withdrawn from the sample valve was subjected to analyses to get 3.0 × 10^8^ cells per mL. The cultures were transferred to second stage of propagation in individual steam-desterilized (45 minutes) fermenter of 3 m^3^ capacity, contained the same essential nutrients of seed fermenter, the fermentation was continued for about 14 hours. In the third stage the yeast cultures from the second stage fermenters were transferred to the propagation tanks of 12 m^3^ capacities with 10 m^3^ working volume containing the same above medium. The fermentation was continued till reducing sugars contents below 1% and ethanol content in the range of 3.2–3.8% (v/v), having 3.0 × 10^8^ cells/mL were prepared for use in industrial fermentation of molasses to ethanol production.

### 2.4. Fermentation Process

Batch culture system was employed for optimization of fermentation parameters for *S. cerevisiae* F-514 strain. The yeast culture was transferred to fermenters having working volume of 65 m^3^. Initially a bed of 25% volume was made by 5 m^3^ yeast culture 3.0 × 10^8^ cells/mL at the bottom of fermenter in molasses medium contained 5-6% sugars, supplemented with the parameters to be optimized, but afterwards feeding of diluted molasses to gave final concentration 18% (w/v) sugars was fed to the fermenters to enable yeast cells to utilize sugars in the molasses for conversion into ethanol. Batch of molasses was adjusted, so that fermenters vessels were filled to 80% working capacity (65 m^3^) and then stayed to ferment for a period of 20 hours to allow the maximum conversion of sugars into ethanol. After 20 hours, the samples collected through sample valves were analyzed for ethanol content, residual sugars, viable cell count, and yeast biomass yield.

### 2.5. Process Optimization

During fermentation stage, all the parameters to be optimized were varied (Urea, diammonium phosphate (DAP), Orthophosphoric acid (OPA), magnesium sulfate and inoculum size). During optimization, temperature and pH were not adjusted.

Cell count optimization was performed by using yeast cell counts 3.0 × 10^8^ cells/mL inoculum.

Varying concentrations of urea, 10, 15, 20, 25, and 30 kg/65 m^3^ were added to the fermentation media in 65 m^3^ working volume fermenter inoculated with 10% v/v yeast inoculum cell counts 300 × 10^6^ cells/mL.

Varying concentrations of ADP (5, 10, 15, and 20 kg/65 m^3^) were added to the fermentation medium under above optimized urea level.

Varying concentrations of orthophosphoric acid (OPA) (5, 10, 15, and 20 kg/65 m^3^) were added to the fermentation media in 65 m^3^ working volume fermenter under optimized urea level.

Combinations of urea, ADP, and OPA, that is, 20, 10, and 5 kg/65 m^3^, respectively, were added to the fermentation medium.

Varying concentrations of magnesium sulfate (5, 10, 15, and 20 kg/65 m^3^) were added to the fermentation medium in 65 m^3^ working volume fermenter under the applied concentrations of urea, ADP, and OPA.

Varying sizes of inoculum (5, 10, 15, 20, and 25 v/v%) were used to inoculate the respective fermentation fermenters under optimized parameters of urea, DAP, OPA, and magnesium sulfate to investigate the effect of inoculum size on ethanol production.

Varying concentrations of molasses under optimized parameters of Urea, DAP, OPA and magnesium sulfate and inoculum size to investigate the effect of molasses concentrations on ethanol production on time.

### 2.6. Analytical Procedures

#### 2.6.1. Determination of Sugar Concentration

The sugar concentration was determined by rapid method. The 5 mL of fermented sample was taken and dissolved in 100 mL of distilled water and mixed with 5 mL of conc. HCL acid and is heated at 70°C for a period of 10 min. The obtained sample was neutralized by adding NaOH and it was prepared to 1000 mL and taken into burette solution. The 5 mL of Fehling A and 5 mL of Fehling B were taken and mixed with 10 to 15 mL of distilled water in a conical flask and methylene blue indicator was added. The conical flask solution was titrated with burette solution in boiling conditions until disappearance of blue color. The sugar concentration was calculated by using the formula given below: Sugar Concentration (gm/L) = [(Dilution factor × Fehling factor)/Titrate value] × 100.

#### 2.6.2. Ethanol Content

Ethanol content of the fermented samples was measured with ebulliometer approved in distillation factories.

#### 2.6.3. Fermentation Efficiency

Fermentation efficiency was calculated as the ethanol yield divided by the theoretical yield multiply by 100.

Cell count was determined using microscope with the help of haemocytometer. Cell viability was checked by using methylene blue indicator. The dead cells were stained with blue indicator while viable cells remained uncolored.

#### 2.6.4. Analysis of Dry Yeast Yield

Crude protein was measured by micro-Kejldahel method [18]. Ash was carried out on dried sample at 105°C, by ignition 3 samples each 50 g in muffle furnace at 800°C for 5 hours, and the residual ash was calculated as % from the dried initial weigh [19]. Cell dry weight was determined using 20 ml samples of the yeast culture collected by centrifugation (10 min at 7500 xg, 4°C) in a pre-weighed dried tube and then washed with 20 ml of distilled water. The tube was dried overnight at 105°C and weighed again.

## 3. Results and Discussion

### 3.1. Effect of Varying Concentrations of Urea (as Nitrogen Source)

Varying concentrations of urea were added as nitrogen supplement for yeast growth (Table 1). Results showed that cell growth and ethanol yield increased with urea addition and 25 kg urea/65 m^3^ fermenter working volume gave maximum ethanol yield (EOH) of 8.4% v/v with remaining sugar (RS) 2.46%, fermentation efficiency (FE) 75.5%, yeast viable cells 2.90 × 10^8^/mL, and dry yeast biomass 11.4 g/L. Nitrogen deficiency slows down yeast growth and the fermentation [20–22], possibly due to the inhibition of the synthesis of protein transporting sugars through the cell membrane to the interior of the cells [23, 24]. It has been shown that adequate nitrogen increases yeast growth provided that the other essential yeast nutrient is not lacking [25–27].

### 3.2. Effect of Varying Concentrations of DAP

Varying concentrations of DAP were used as phosphorus and supplementary nitrogen source to promote yeast growth and increase ethanol production (Table 2). At DAP concentration of 15 kg/fermenter 65 m^3^ working volume, *S. cerevisiae* F-514 produced 8.9% (v/v) ethanol with RS, 1.66%, FE 80.1% final cell count 3.10 × 10^8^/mL, and dry yeast cells 11.8 g/L.

### 3.3. Effect of Varying Concentrations of OPA

Phosphate limitation has been shown to affect cell growth and biomass formation as well as directly affecting fermentation rate [28, 29]. Varying concentrations of OPA were used as phosphorus source under the optimum amount of urea to promote yeast growth and increase ethanol production (Table 3). At OPA concentration of 15 kg/fermenter 65 m^3^ working volume, *S. cerevisiae* F-514 produced 9.1% (v/v) ethanol with FE 81.8%, remaining sugars, 1.32, final cell count 3.40 × 10^8^/mL, and dry yeast cells 11.7 g/L.

### 3.4. Effect of Combination of Urea, of DAP and OPA

Date presented in Table 4 Showed that combination from 20, 10, and 5 kg of urea. DAP and OPA, respectively/65 m^3^ medium more suitable for ethanol yield comparable with urea plus DAP or urea plus OPA (Tables 2 and 3) as produced 9.3% (v/v) ethanol with FE 83.6%, remaining sugars, 1.32, final cell count 3.45 × 10^8^/mL and dry yeast cells 12.1 g/L. Nitrogen and phosphorus are the main nutritional requirements for the yeast growth and maximum ethanol production efficiency. Although molasses contains most of the nutrients required for yeast growth, generally nitrogen and phosphate are added to enhance yeast growth and ethanol production [30]. For optimum yeast efficiency in molasses medium, urea was used as nitrogen source and OPA was used as phosphate source. Phosphorus has the major role in the glycolysis cycle in the yeast cell. Extensive studies were previously performed to optimize the nitrogen and phosphorous sources and other supplements [31]. Higher ethanol production has also previously been reported with urea, phosphoric acid, making the process very economical [27]. Phosphate limitation has been shown to affect cell growth and biomass formation as well as directly affecting fermentation rate [28, 29].

### 3.5. Effect of Varying Concentrations of Magnesium

Varying concentrations of magnesium sulfate were supplement under the above optimized levels of urea, DAP and OPA for yeast growth (Table 5). Results showed that cell growth and ethanol yield and fermentation efficiency increased with magnesium sulfate addition and 10 kg concentration gave maximum ethanol content of 9.6% (v/v) ethanol with remaining sugars, 1.32, final cell count 3.60 × 10^8^/m, and yeast yield 12.4 g/L. Deficiencies and imbalances in minerals and cations serving as cofactors for glycolytic and other enzymatic reactions can result in fermentation arrest [32]. Magnesium plays a key role in metabolic control, growth and cell proliferation, glycolytic pathway, and subsequently ethanol production [33].

### 3.6. Effect of Varying Inoculum Size

Ethanol yield and production of coproducts have a major relationship during ethanol fermentation. Extensive studies have been carried out to investigate the effect of yeast inoculation rate to help the yeast cells overcome the bacterial cells on the basis of size and number. Effect of varying inoculum sizes on ethanol yield was studied under optimized parameters, urea 20 kg, DAP 10 kg, OPA 5 kg, and magnesium sulfate 10 kg/65 m^3^ working volume fermenter. Maximum ethanol content was found at an inoculation rate of 20% v/v. Results have shown that at 20% inoculation rate, ethanol content was 9.8% (v/v) (Table 6). In brewing, higher yeast inoculation rates cause attenuation to initiate the process more rapidly and reduce viability losses that occur immediately after pitching. In a previous study, the ethanol yield increased with increasing inoculum size and yield of methanol or aldehyde was the lowest at inoculum size above 30% [34] and gave optimum ethanol content 9.8% v/v. The remaining sugars were 1.22%, final viable cell count 3.60 × 10^8^/mL, and yeast yield 13.2 g/L.

### 3.7. Effect of Varying Sugarcane Molasses Concentrations

Varying doses of sugar cane molasses contained varying sugars concentration were applied to study the effect of sugar level in fermentation medium on the ethanol yield on time under the above optimized levels of urea, DAP. OPA, magnesium sulfate and inoulum size (Table 7). Results show that most suitable sugars concentration for ethanol production by* S. cerevisiae *F-514 was 18% (w/v) gave high ethanol yield 9.8% (v/v), FE 88.1%. RS 1.22% and applied low or above other sugar concentrations not economic. Increase in medium sugar level is believed to affect the relative proportion of total medium sugar converted to alcohol [17, 35, 36]. The decline in yeast ethanol productivity at high medium sucrose levels as observed in this study is in close agreement with the finding of several other researchers of the *Saccharomyces *genus in medium of high osmotic pressures [35, 37, 38].

### 3.8. Chemical Composition of Yeast Yield

The chemical composition of the yeast on basis of dry weight was protein 34.6% and ash 8.2%. Our study was applied in distillery factory distilled about 1600 m^3^ daily producing about 20 tons of fodder yeast. Yeasts are a rich source of protein and B-complex vitamins. They have been used successfully as a complementary protein source in fish diet [39]. Also, they have been used as a supplement in animals feed to compensate for the amino acid and vitamin deficiencies of cereals and are recommended as a substitute for soybean oil in diets for fowl [40], and flavor enhancers can be produced from yeasts [41]. In addition, they are considered a cheaper dietary supplement as they are easily produced on an industrial level [42].

The results of our study showed that under optimum conditions. However, optimization of process parameters improved ethanol production by the local yeast strains of *S. cerevisiae* F-514 in Egyptian Distillation Factories without needing to cooling system that make ethanol production more economic. The obtained ethanol concentration in this study was higher than that obtained by other workers studied the optimization fermentation conditions for producing ethanol from cane molasses under industrial scale by batch or fed batch fermentation using other different yeast strains, as it were 7.9% (v/v) Abd El Fattah et al. [6], 8.6% (v/v) Arshad et al. [34] and 8.2% (v/v) Mukhtar et al. [43].

## Figures and Tables

**Table 1:** Effect of varying concentrations of urea (as nitrogen source) on ethanol production by *S*. *cerevisiae *F-514 using sugarcane molasses.

Urea kg/65 m^3^	Initial pH	EOH% v/v	RS%	FE%	Final pH	Viable yeast cells ×10^8^	Yeast yield (g/L)
10	4.6	7.8	3.11	70.1	4.9	2.55	10.4
15	4.6	8.1	2.85	72.8	5.0	2.75	10.8
20	4.7	8.3	2.60	74.6	5.1	2.90	11.4
25	4.7	8.4	2.46	75.5	5.2	2.90	11.4
30	4.8	8.2	2.66	73.7	5.4	2.85	11.2

**Table 2:** Effect of varying concentrations of DAP on ethanol production by *S*. *cerevisiae* F-514 using sugarcane molasses.

DAP kg/65 m^3^	Initial pH	EOH% v/v	RS%	FE%	Final pH	Viable yeast cells ×10^8^	Yeast yield (g/L)
5	4.7	8.5	2.16	76.4	5.2	2.95	11.1
10	4.7	8.7	1.96	78.2	5.2	3.00	11.4
15	4.8	8.9	1.66	80.1	5.3	3.10	11.8
20	4.8	8.9	1.64	80.1	5.4	3.10	11.6

**Table 3:** Effect of varying concentrations of OPA on ethanol production by *S*. *cerevisiae* F-514 using sugarcane molasses in batch culture.

OPA kg/65 m^3^	Initial pH	EOH% v/v	RS%	FE%	Final pH	Viable yeast cells ×10^8^	Yeast yield (g/L)
5	4.7	8.6	1.72	77.3	4.9	3.00	11.2
10	4.7	8.8	1.56	79.1	4.8	3.20	11.4
15	4.8	9.1	1.32	81.8	4.8	3.40	11.7
20	4.8	9.0	1.46	81.7	4.6	3.20	11.6

**Table 4:** Effect of combination of urea, of DAP, and OPA on ethanol production by *S*. *cerevisiae *F-514 using sugarcane molasses.

kg/65 m^3^	Initial pH	EOH% v/v	RS%	FE%	Final pH	Viable yeast cells ×10^8^	Yeast yields (g/L)
Urea 20*	4.7	8.3	2.6	74.6	51	2.90	11.4
*+DAP10**	4.7	8.7	1.74	78.2	5.2	3.15	11.8
**+OPA 5	4.8	9.3	1.32	83.6	5.1	3.45	12.1

**Table 5:** Effect of varying concentrations of magnesium sulfate on ethanol production by *S*. *cerevisiae *F-514 using sugarcane molasses in batch culture.

Magnesium sulfate kg/65 m^3^	Initial pH	EOH% v/v	RS%	FE%	Final pH	Viable yeast cell ×10^8^	Yeast yield (g/L)
5	4.7	9.5	1.40	85.4	4.8	3.60	12.4
10	4.7	9.6	1.32	86.3	4.8	3.60	12.4
15	4.8	9.6	1.36	87.1	5.0	3.60	12.2
20	4.8	9.5	1.40	85.4	5.0	3.55	12.1

**Table 6:** Effect of varying inoculum size on ethanol production by *S*. *cerevisiae *F-514 using sugarcane molasses.

Inoculum size% (v/v)	Initial pH	EOH% v/v	RS%	FE%	Final pH	Viable yeast cell ×10^8^	Yeast yield (g/L)
5	4.7	9.4	1.71	84.5	4.8	3.15	12.2
10	4.7	9.6	1.36	86.3	4.8	3.60	12.4
15	4.8	9.7	1.28	87.2	5.0	3.75	13.1
20	4.8	9.8	1.22	88.1	5.2	3.80	13.2
25	4.8	9.6	1.64	86.3	5.2	395	13.6

**Table 7:** Effect of varying sugarcane molasses concentrations on time on ethanol production by *S. cerevisiae* F-514.

Sugar% (w/v)	Fermentation time (hrs)										FE%
	16		18		20		22		24		
	EOH% (v/v)	RS%	EOH% (v/v)	RS%	EOH% (v/v)	RS%	EOH% (v/v)	RS%	EOH% (v/v)	RS%	
16	8.2	1.34	8.6	1.32	8.6	1.22	8.5	1.22	8.5	1.22	86.9
17	8.7	1.46	8.9	1.28	9.1	1.28	9.3	1.28	8.8	1.28	86.5
18	9.4	1.68	9.9	1.22	9.8	1.22	9.8	1.22	9.8	1.22	88.1
19	9.2	1.86	9.4	1.56	9.7	1.96	9.8	1.74	9.8	1.74	83.4
20	8.9	3.45	9.4	2.84	9.7	2.96	9.8	2.86	9.8	2.86	79.2
21	8.2	4.65	8.4	3.55	9.4	3.20	9.6	2.20	9.6	2.20	73.9
22	7.8	6.24	8.2	5.42	8.6	3.22	9.2	3.24	9.4	3.24	69.1
